# Metabolic fate of orally administered cGMP in rats

**DOI:** 10.1186/2050-6511-14-S1-P39

**Published:** 2013-08-29

**Authors:** Elaine Liong, Christopher Graul, Samuel Rivers, Marco Kessler, Gerhard Hannig, Inmaculada Silos-Santiago

**Affiliations:** 1 Ironwood Pharmaceuticals, Cambridge, MA 02142, USA

## Background

Activation of membrane guanylate cyclase type C (GC-C) on the luminal surface of the intestinal epithelium by GC-C agonists results in an increase in both intracellular and extracellular levels of cyclic guanosine monophosphate (cGMP). Elevation in intracellular cGMP results in the secretion of chloride and bicarbonate anions into the intestinal lumen through the activation of the cystic fibrosis transmembrane conductance regulator (CFTR) ion channel. Physiologically, this results in increased intestinal fluid and accelerated transit. Furthermore, an accompanying increase in the luminal and submucosal concentration of cGMP is observed as intracellular cGMP is transported out of epithelial cells. In animal models, decreased activity of pain-sensing afferent fibers is likely mediated by increased extracellular cGMP in the submucosa [1]. Compared to the pharmacological effects elicited by the increase in cGMP, less is known about the distribution and metabolic fate of this second messenger that is transported out into the luminal space.

## Results and conclusion

We used radiolabeled 14C-cGMP to assess the presence and metabolism of cGMP following oral administration in different regions of the gastrointestinal tract in rats, as well as in plasma and urine. In tracking the levels of 14C-cGMP and its metabolites we developed a better understanding of the fate of orally dosed cGMP in the intestinal tract, which has implications for the mechanism of action of cGMP in decreasing gastrointestinal pain. Specifically, we hypothesize that cGMP may act on intestinal afferent fibers while travelling along the small intestine whose projections converge on second order neurons that also receive input from colonic afferents, thus affecting their function. In this context, we showed that more than 50% of the dosed cGMP is available in the small intestine 2 hours after dosing. Little/no 5’GMP or other metabolites were detected in the luminal contents. In contrast, 40-50% of cGMP metabolites were found in tissue samples.
